# Contributions to Obstetric Jurisprudence

**Published:** 1858-11

**Authors:** 


CONTRIBUTIONS TO OBSTETRIC JURISPRUDENCE.—Commencing with criminal abortion in our January number, Dr. Horatio R. Storer will present our readers with a series of important papers on obstetric jurisprudence, to which no one in this country has paid so much attention as himself, and which in point of fact has never been, as such, touched upon. Dr. Storer, Senior, three years since was the first to call attention to criminal abortion in an introductory lecture in Harvard University, and a similar address was delivered by Prof. Hodge in the University of Pennsylvania. In February, 1857, Dr. H. R. Storer presented the subject to the Suffolk District Medical Society, and was appointed one of a committee to report upon it, which he did in a very able and forcible manner. Dr. Storer has taken a very decided and praiseworthy position in the matter, and we hope these papers will serve to awaken the attention of and prepare the minds of the profession for the report on criminal abortion in this country, which Dr. Storer is to make to the American Medical Association.

